# GMA Annual Conference 2016 in Bern – Conference Report

**DOI:** 10.3205/zma001079

**Published:** 2017-02-15

**Authors:** Sissel Guttormsen, Daniel Bauer, Jan Breckwoldt, Sören Huwendiek, Kai Schnabel, Christian Schirlo

**Affiliations:** 1 Universität Bern, Institut für Medizinische Lehre, Bern, Schweiz; 2 Universität Zürich, Medizinische Fakultät, Koordinator Studiendekanat, Zürich, Schweiz; 3 Universität Zürich, Med. Fakultät, Dekanat, Stabsleiter, Zürich, Schweiz

## Introduction

Under the motto “Innovative Together” and for the first time in 15 years, the annual meeting of the Association for Medical Education (GMA) was held in Switzerland from 14 to 17 September 2016. More than 450 participants made their way to Bern and contributed to a lively event, by presenting their projects, moderating, discussing, asking questions and providing answers. 

Under the patronage of the two conference presidents Sissel Guttormsen (Bern) and Christian Schirlo (Zurich), 274 scientific papers and 36 workshops tackled various projects and questions of education, further education and CPD in human, dental, veterinary medicine and academic health professions. 

All contributions had previously been critically reviewed by two reviewers. The abstracts for all scientific contributions were published via the German Medical Science (GMS) portal for the conference and are available at http://www.egms.de/dynamic/en/meetings/gma2016/index.htm.

Various welcome speakers from the Swiss educational landscape as well as exhibitors provided valuable contributions and together with the sponsors and our cooperation partners (Federal Office of Public Health of the Federal Department of Home Affairs and the Robert Bosch Foundation), provided a framework for the conference. The program is available online at http://box.iml.unibe.ch/gma-programm/GMA2016_Prog.pdf. 

## Program

The motto “Innovative Together” was a central theme of the annual conference. The program design was made *Innovative* by incorporating more modern formats in addition to the “classical” formats such as short and poster presentations. In the so-called “flipped contributions”, based on the Flipped Classroom Method [1], speakers were able to make informational material available in advance, in order to allow the sessions to be fully utilized for deep discussions. The format of “demo contributions” was also introduced to give space for demonstrations of software or learning tools. And for the first time at a GMA annual conference, the format “Fringe/Weird Stuff” was tested. This format is inspired by AMEE, which describes fringe contributions as “*an opportunity to take a different perspective and a new, perhaps provocative or idiosyncratic approach to health education. [...] The emphasis is on creativity, performance and involvement of the audience”* [https://www.amee.org/conferences/amee-2016/abstracts#amee-fringe].

The element of *Togetherness* appeared, for example, in the innovative implementation of the keynotes. Thus keynote speeches were not given by a single speaker as is common but instead by a team of two, speaking jointly and sharing discussion time. In addition, keynote speakers were asked beforehand to incorporate specific measures into their plenary lectures in the form of structured interactions to encourage audience participation and thus to explicitly apply didactic principles of large group teaching [2]. Afterwards participants had the chance to send open questions (via SMS, browser, app or Twitter) to the speakers via Poll Everywhere [https://www.polleverywhere.com/], which were subsequently discussed in a “Meet the Experts” round. The plenary lectures focused on different aspects of competency orientation and ‘Entrustable Professional Activities’, the added value of narratives in learning, the need for innovation in our educational systems, the interplay of patient centering and assessment and not least interprofessionalism - joint learning and working together in medicine. The keynote lectures were recorded and are accessible in the member area of the conference web pages at https://gma2016.de/programm.html. The main program of 145 short presentations (including eight flipped contributions), 120 posters, four fringe and five demo contributions and numerous workshops tackled topics such as education research, assessment, curriculum development, interprofessionalism, further education and much more, and resulted in reflection, lively debates and exchange of experiences.

During the evening reception in the historic Kornhauskeller in the old town of Bern, the GMA award winners were also honoured: 

### GMA Project Prize for the Development of Teaching 2016 (joint first place):

- Ronny Lehmann: *Blended Learning with Virtual Patients and Skills Labs to Improve the Learning of Clinical Practical Skills*
- Sabine Salloch, Ina Otte, Caroline Ruiner, Jochen Vollmann: *Medicine meets Economics. An interdisciplinary expedition to the hospital*
- Fabian Stroben, Katja Anne Dannenberg, Tobias Deselaers, Dorothea Eisenmann, Julia Freytag, Leopold Vorwerk: *“Night shift: Are you ready?” - a simulated night in the emergency room of the Charité Learning Center*

#### GMA-Prize for innovative teaching projects 2016” (joint first prize):

- Paul von Poellnitz, Merit Stenzel, Irmgard Streitlein-Böhme: *Seminar on Learning Methods*
- Konrad Meissner, Christiane Reppenhagen, Maud Partecke: *Increasing patient safety by integrating interprofessional Human Factor Training into the training of health care professionals*

#### Publication Prize of the GMS Journal for Medical Education 2016:

- Jan Kiesewetter, Martin Fischer: *The Teamwork Assessment Scale: A Novel Instrument to Assess Quality of Undergraduate Medical Students' Teamwork Using the Example of Simulation-based Ward-Rounds *[3]

In addition, an exceptional **“Innovative Together Prize”** was also awarded by the organisers of the annual conference for the contribution to the scientific program (excluding the plenary lectures) which best reflected the spirit of the conference motto. Based on feedback gathered from the conference participants, the prize committee ultimately decided that this was the contribution in which representatives of all North Rhine-Westphalian medical faculties jointly and innovatively explored the possibilities of the fringe format to shed light on the pitfalls and challenges of networks:

- Angelika Fritz Hiroko, Christian Thrien, Jörg Reissenweber, Anke Adelt, Andrea Rietfort, Bernhard Steinweg, Gabriele Campe, Linn Hempel, Franz-Bernhard Schrewe, Tim Peters: *„Live Networks for Dummies“* [4].

Of course numerous meetings of the GMA committees as well as the annual general assembly were also held. They also presented an opportunity for interested conference attendees to gather information about the work of the respective committees. Please refer to the minutes of each session.

In summary, the feedback and the evaluation show that the conference and the numerous innovations were well received. We recommend further reflection on the conference formats and to provide didactically sound and innovative formats at the annual GMA conference.

## Thanks and outlook

We would like to thank all those who have made the annual conference possible through their enthusiasm and tireless efforts. We are also very grateful to the many staff members of the Institute of Medical Education (IML) who were responsible for running the event and who have gone to great lengths to make this this large event happen. The good cooperation between the teams from Bern and Zurich was enriching throughout, both before, during and after the conference. In addition, we would like to thank the GMA Executive Board and Mrs Herrmannsdörfer of the GMA office, the GMS employees in Cologne and many others.

The next annual conference will take place 20 to 23 September 2017 in Muenster and will once again be held as a joint conference of the German Association for Medical Education (GMA) and the Working Group for the Further Development of Teaching in Dental Medicine (AKWLZ) under the motto “Realization of Education”. Further information is available at www.gma2017.de. 

## Competing interests

The authors declare that they have no competing interests.
